# Structural Properties and Phase Stability of Primary Y Phase (Ti_2_SC) in Ti-Stabilized Stainless Steel from Experiments and First Principles

**DOI:** 10.3390/ma12071118

**Published:** 2019-04-04

**Authors:** Deli Zhao, Yu Zhou, Jiangyu Fan, Tianyu Liu, Yihong Nie, Wantang Fu, Zhiqing Lv

**Affiliations:** 1 State Key Laboratory of Metastable Material Science and Technology, Yanshan University, Qinhuangdao 066004, China; zhaodel@gmail.com (D.Z.); wtfu@ysu.edu.cn (W.F.); 2 Key Laboratory of Advanced Forging & Stamping Technology and Science (Yanshan University), Ministry of Education of China, Qinhuangdao 066004, China; 18712727819@163.com (Y.Z.); 18716027532@163.com (J.F.); YDliutianyu521@163.com (T.L.); 3 China First Heavy Industries, Qiqihar 161042, China; nie.yh@cfhi.com

**Keywords:** phase stability, Ti-containing steels, structural evaluation, electronic structure, first principles

## Abstract

The morphology and microstructural evaluation of Y phases in AISI 321 (a Ti-stabilized stainless steel) were characterized after hot deformation. The electronic structure and phase stability of titanium carbosulfide were further discussed by first-principle calculations. It was found that Y phases, like curved strips or bones in AISI 321 stainless steel, mostly show a clustered distribution and are approximately arranged in parallel. The width of the Y phase is much less than the length, and the composition of the Y phase is close to that of Ti_2_SC. Y phases have exceptional thermal stability. The morphology of Y phases changed considerably after forging. During the first calculations, the Ti_2_SC with hexagonal structure does not spontaneously change into TiS and TiC; however Ti_4_S_2_C_2_ (Z = 2) can spontaneously change into the two phases. The Ti–S bonds are compressed in Ti_4_S_2_C_2_ cells, which leads to poor structural stability for Ti_4_S_2_C_2_. There is a covalent interaction between C/S and Ti, as well as an exchange of electrons between Ti and S/C atoms. Evidently, the mechanical stability of Ti_4_S_2_C_2_ is weak; however, Ti_2_SC shows high stability. Ti_2_SC, as a hard brittle phase, does not easily undergo plastic deformation.

## 1. Introduction

Stainless steels are the most common construction materials used by the petrochemical, chemical, and fertilizer industries as well as power plants (especially in nuclear and solar power plants). Stainless steels are selected mainly for a good combination of mechanical, oxidation and corrosion resistance properties [1,2,3,4]. AISI 321, a stabilized stainless steel containing Ti, has potential applications as heat exchangers in nuclear power plants because of its relatively moderate mechanical strength, impact toughness (even at low temperatures), and excellent resistance to corrosion and oxidation [4,5,6,7]. The Ti-stabilized stainless steels (AISI 321, AISI316-Ti, etc.) and Nb-stabilized stainless steels (AISI 347) have a stronger combining capacity with carbon than chromium, thus resulting in titanium carbide or niobium carbide, etc. The formation of titanium carbide or niobium carbide is beneficial to avoid chromium impoverishment after the precipitation of chromium carbide [1,5,6,7,8].

Sulfur, a harmful element in steel, is reduced to the lowest possible level during steelmaking. Generally, the sulfur content of stainless steels is less than 0.03 mass%. Although the sulfur content in stainless steels is very low, titanium sulfides (TiS or τ-Ti_2_S etc.) or titanium carbosulfide (Y phases, Ti_4_S_2_C_2_ or Ti_2_SC etc.) is an important primary phase observed in titanium-containing steels (AISI 321), nickel-based superalloys and Fe-based superalloys etc. [9,10,11,12,13,14,15], especially for large castings and forgings. The characterization and structure of sulfides in titanium-containing steels have been the focus of discussion [15,16,17]. Although Ti_2_S with a hexagonal structure has been found to exist in steel [16], the existence form of this phase in steel was questioned, and Ti_4_S_2_C_2_ was likely identified inaccurately [15,17]. Wilson and Chen [15] observed the hexagonal-structured iron titanium sulfide (Ti_[1−x]_Fe_x_S) in Ti-containing low manganese steel. Casa and Nileshwar [12] showed that the Y phase in 321 steel was a hexagonal-structured carbosulfide with a composition of approximately Ti_1−4_CS. Maloney et al. [14] discussed the effects of sulfide type on the fracture toughness of HY 180 steel, and pointed out that the fracture toughness of Ti-modified HY180 steels obviously increases due to the formation of Ti_2_SC, replacing MnS and La_2_O_2_S. The evaluation and stability of these primary phases (TiS, Ti_4_S_2_C_2_ or Ti_2_SC etc.) can strongly influence the properties of materials and the subsequent processing, which needs to be studied deeply and carefully.

This work was to study the morphology of titanium carbosulfide (Y phase) in AISI 321 stainless steel. The morphology of the Y phase was characterized after hot deformation. The structural properties and phase stability of titanium carbosulfide were further discussed using first-principle calculations.

## 2. Experimental Procedure and Calculation Details

### 2.1. Experimental Method

**Figure 1:**
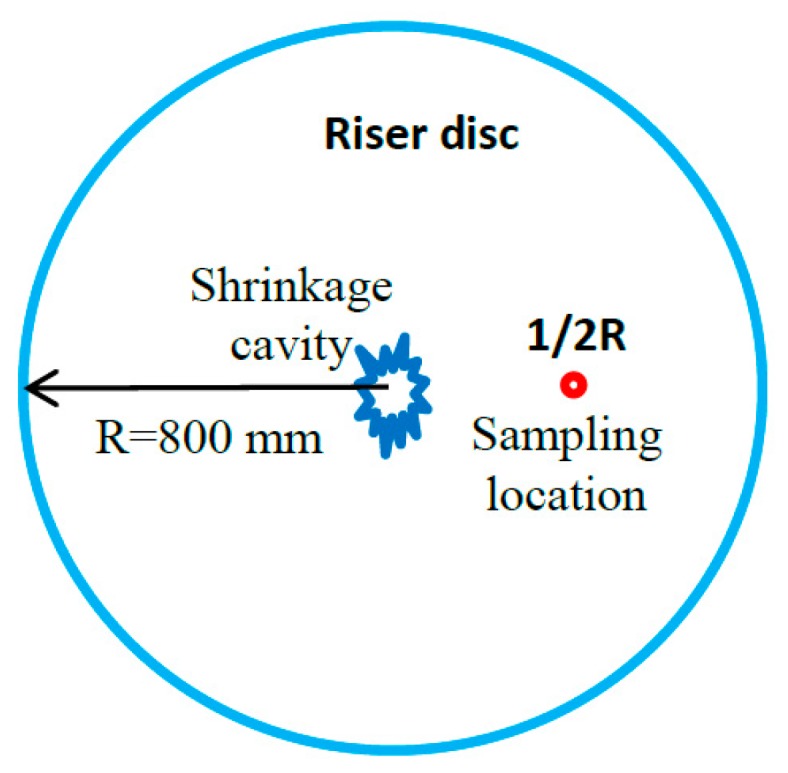
Schematic diagram of sampling location.

The experimental material was AISI 321 austenitic stainless steel. The chemical composition (in mass%) is: 0.076C, 17.82 Cr, 11.09 Ni, 2.05 Mn, 0.4 Ti, 0.5 Si, 0.2 Mo, 0.2 Cu, 0.008 S, 0.03 P, and balance Fe. The experimental samples were cut from a riser disc in the riser part of a 93 t ingot, as shown in Figure 1. The 93 t ingot was obtained by double vacuum smelting method including of vacuum electroslag melting (VOD) and vacuum gas storage (VD), and the diameter of the ingot was about 2000 mm. The riser disc, with a thickness of about 105 mm, was cut from 300 mm to the top of the riser, and the radius of the disc was about 800 mm. The size of the forging sample was ϕ60 mm × 105 mm, and the forging samples were from the 1/2R of the riser disc.

**Figure 2:**
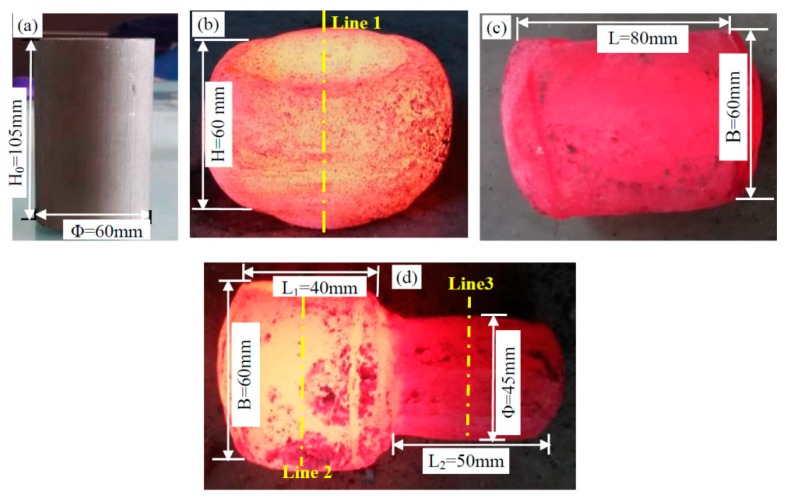
The forging process of the sample: (**a**) forging stock before deformation, ϕ60 mm × 105mm; (**b**) first step-upsetting deformation, upsetting ratio K_0_ = 1.75; (**c**) second step-squaring process, forging ratio K_L1_ = 1.37; and (**d**) third step-stretching and chamfering-rounding, forging ratio K_L2_ = 3.11.

The sample, after homogenizing annealing at 1290 °C for 24 h, was forged at 1200 °C. The forging process included three steps: one upsetting deformation and two squaring processes, as shown in Figure 2. The upsetting ratio (H_0_/H) was K_0_ = 1.75 in the first step, the forging ratio was K_L1_ = 1.37 in the second step, and the forging ratio was K_L2_ = 3.11 in the third step. The samples were air cooled after forging. The forging processes were carried out on a 500 t hydraulic machine. The working speed was about 6–10 mm/s, which was in accordance with the industrial production of large forgings.

Morphological and composition analyses were conducted on a Zeiss-Sigma 500 scanning electron microscope (SEM, Carl Zeiss, Oberkochen, Germany) with energy dispersive spectrometry (EDS) at an acceleration voltage of 20 kV. The samples for observation were not etched after mechanical polishing. The microstructural observation locations of the forging samples are the center positions of the cross-sections, which were cut along yellow lines (line 1, line 2 and line 3), as shown in Figure 2.

### 2.2. Calculation Details

The density functional calculations are carried out with the CASTEP code [18,19]. The interactions between the ion and electrons are modeled by ultrasoft pseudopotentials of the Vanderbilt type [20]. The spin-polarized Perdew-Burke-Ernzerhof (PBE) function within the generalized gradient approximation (GGA) was used to evaluate the exchange-correlation potential [21,22]. It was expanded in a plane wave basis for the Kohn-Sham one-electron states, and the energy cutoffs were set up to 400 eV. Using a 2 × 2 × 8 k-point grid of the Monkhorst-Pack scheme, the energy calculations were made in the first irreducible Brillouin zone [23]. The convergence criteria for each calculation were set to ultra-fine quality; the energy, maximum force on the atom, maximum displacement between cycles and tolerance for the stress concentration factor were set to: 10^−5^ eV/atom, 0.1 eV/nm, 5 × 10^−5^ nm, and 10^−6^ eV/atom, respectively.

## 3. Results and Discussion

### 3.1. Morphology of Y Phase in Cast Ingot and Annealing Structure

**Figure 3:**
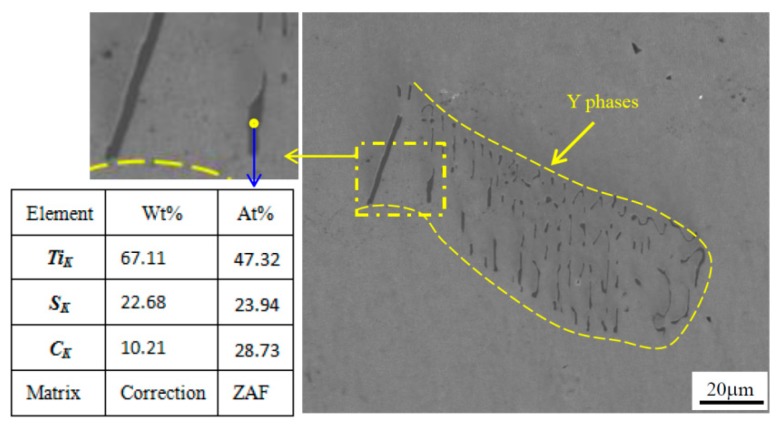
SEM image and EDS analysis of Y phases in AISI 321 form the cast ingot.

From the analyses of microstructures in cast ingot, a small number of Y phases can be found to be distributed in AISI 321 stainless steel. These phases are primary phases in the solidification process of steel liquid. Figure 3 shows the SEM image and EDS analysis of Y phases in the cast ingot. Y phases in AISI 321 stainless steel are like curved strips or bones, which mostly show clustered distribution and an approximately parallel arrangement. The morphology is similar to that of M2C carbides in high-speed steel [24]. The width of Y phases mainly ranges from 0.5 to 2 μm; however, the length varies from a few microns to tens of microns, with the longest one being about 60 μm. The EDS analyses of these Y phases were given, and the chemical compositions of these phases mainly included Ti, S, C and a small amount of Fe, Cr, etc. Figure 3 shows the EDS analysis of one point in the SEM image (microelements, such as Fe, Cr, etc., were removed or changed to the content of Ti), and the composition of Y phase is close to that of Ti_2_(SC). 

**Figure 4:**
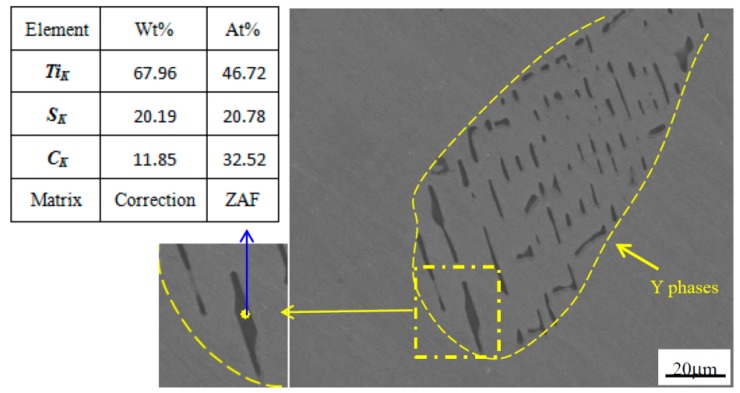
SEM image and EDS analysis of Y phase in AISI 321 after homogenizing annealing at 1290 °C for 24 h.

Figure 4 shows the SEM image and EDS analysis of the Y phase in AISI 321 after homogenizing annealing at 1290 °C for 24 h. After homogenizing annealing (1290 °C for 24 h), there was no significant change in the distribution and metallographic characteristic of the Y phase, which is similar to that in the cast ingot. The arrangement of these Y phases is more regular than that in cast ingot. The width of Y phases mainly ranges from 0.5 to 2 μm, which is consistent with the casting structure. The average length of Y phases decreases slightly. The EDS analyses indicates that the contents of these Y phases change slightly, and the chemical compositions of these phases mainly included Ti, S and C and a small amount of Fe, Cr etc. Figure 4 shows the EDS analysis of one point in the SEM image, and the composition of Y phase is close to Ti_2_(SC). The annealing temperature 1290 °C is the upper limit for the homogenization of AISI 321 steels. After homogenizing annealing at 1290 °C for 24 h, Y phases are basically not dissolved, which indicates that Y phases exhibit high thermal stability. 

### 3.2. Morphology of Y Phase after Forging

**Figure 5:**
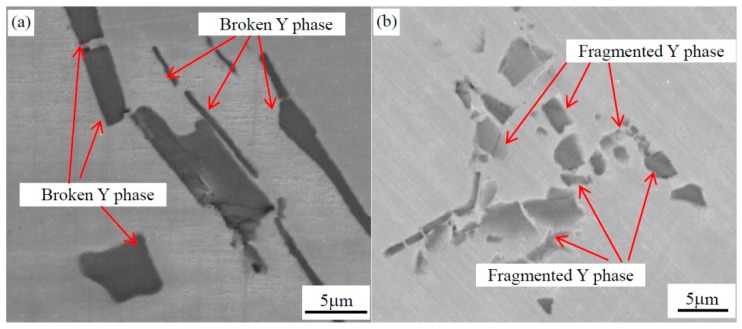
SEM images of the Y phase in AISI 321: (**a**) after second step forging, K_L1_ = 1.37; and (**b**) after third step forging, K_L2_ = 3.11.

Forging can improve the cast structure and refine grains. The evaluation of Y phases is characterized after forging, which includes one upsetting deformation and two squaring processes. The forging process in this work was presented in Section 2, as shown in Figure 2. The SEM analyses of Y phases indicate that Y phases were broken and fragmented during the forging process. Figure 5 shows the SEM images of Y phase after the second step forging and third step forging. From Figure 5a, Y phases, especially for longer ones, are clearly broken up and change to several short-rods from one longer strip.

The shape of Y phases changes to short-rod or particles from curved strips after forging. Y phases were further shattered and some short rods changed to particles from Figure 5b. This broken and fragmented process of Y phases is like the spheroidizing process of the cementite in pearlitic steels [25,26,27]. The degree of fragmentation increases with the increase in forging deformation for Y phases and the sizes of Y phases decrease with increasing deformation.

### 3.3. Size Distribution of Y Phases

In order to analyze the morphologic evaluation of the Y phase with deformation, the size distribution of Y phases was discussed. The average size of the Y phase in the experimental steel after heat treatments and forging was calculated. At the polished plane, the area of a 2-dimensional section was measured and calculated for each particle. The particle was converted to a circle, and an equivalent diameter of the particle was obtained. For each specimen, several micrographs were taken with magnifications from at least 1000 to 5000. For each kind of sample, the total number of measured particles is more than 500. 

**Figure 6:**
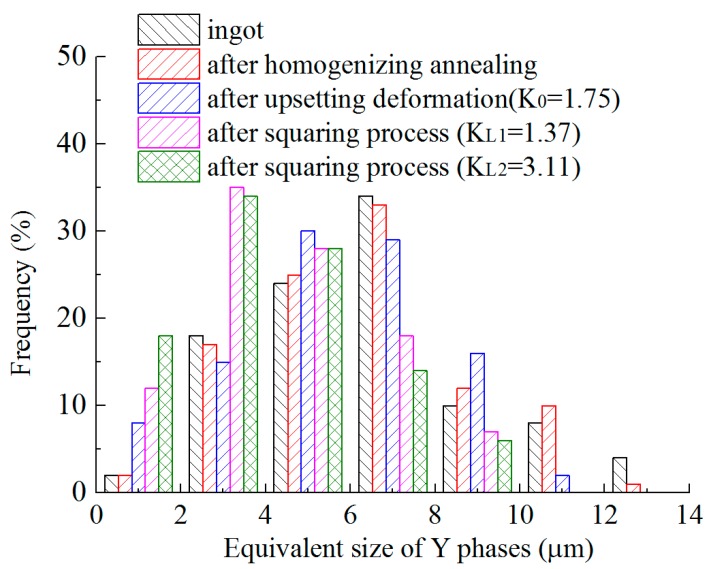
Distribution of the sizes of Y phases after the heat treatment and forging processes.

Figure 6 shows the distribution of the equivalent sizes of Y phases after the heat treatment and forging processes. The biggest equivalent size of Y phases is about 13 μm in the ingot. The equivalent sizes are mainly between 3 and 9 μm, and the frequency of the equivalent sizes is about 86%. The distribution of the equivalent sizes of Y phases in the samples after homogenizing annealing is similar to that in the ingot. The frequency of the equivalent sizes from 3 to 9 μm is about 87% in the samples after homogenizing annealing. The smaller Y phases increase after forging. The largest equivalent size of Y phases is about 11 μm in the samples after the upsetting deformation (the first step). The frequency of equivalent sizes less than 7 μm is about 82% after the upsetting deformation. The biggest equivalent size of the Y phases is about 9 μm in the samples after the second step deformation, and the frequency of the equivalent sizes less than 7 μm is about 93%. The frequency of equivalent sizes less than 7 μm is about 94% in the samples after the third step deformation. The number of smaller Y phases increases with increasing deformation, while that of the larger ones decreases. 

**Figure 7:**
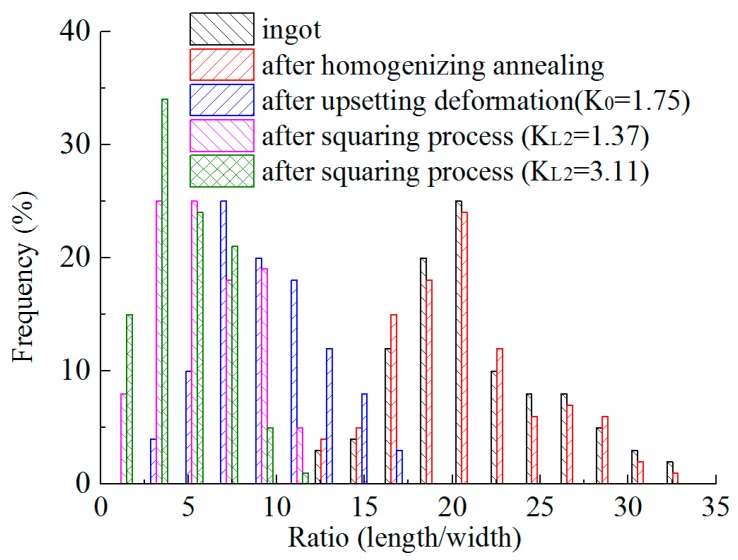
Distribution of the ratios (length/width) of the Y phases after the heat treatment and forging processes.

If the length of the Y phase is evidently larger than the width, the morphologic evaluation of the Y phase cannot be shown clearly only by the equivalent sizes of Y phases. The length-width ratios of Y phases are further discussed. Figure 7 shows the distribution of the length/width ratios of Y phases after the heat treatment and forging processes. The ratios of Y phases in the ingot are mostly between 17 and 23, and the frequency is about 67%. The length-width ratios of Y phases in AISI 321 after homogenizing annealing are similar to that in the ingot. The frequency of the ratios from 17 to 23 is about 69% after homogenizing annealing. The ratios of Y phases are no less than 13 in the samples with these two states. After forging deformation, the length-width ratios of Y phases obviously decrease. The biggest value decreases to about 17 after the first step deformation, and the biggest values decrease to about 11 after the second and third step deformation. The ratios of Y phases in AISI 321 after the upsetting deformation (the first step) are mostly between 5 and 13, and the frequency is about 85%. The ratios of Y phases in the sample after the second step mainly range from 3 and 9, with a frequency of about 87%. The ratios of Y phases in the sample after the third step mainly are from 1 and 7, and the frequency is about 94%. With the increase in deformation, the values of length and width are close to each other for Y phases.

### 3.4. Crystal Structure and Phase Stability

**Figure 8:**
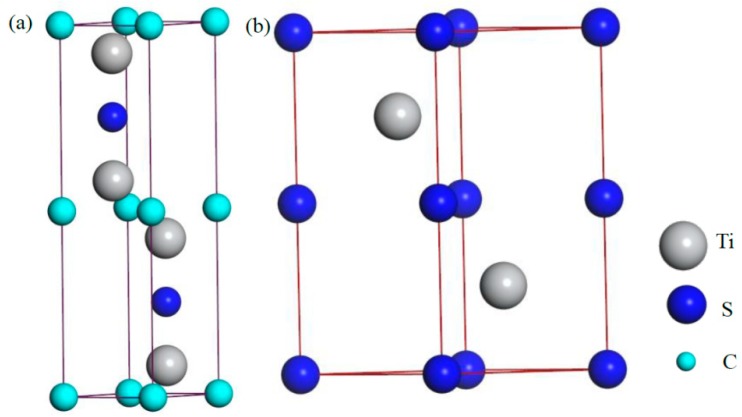
Crystal structure of Ti_4_S_2_C_2_ and Ti_2_S_2_: (**a**) Ti_4_S_2_C_2_ with a hexagonal structure, Z = 2; and (**b**) Ti_2_S_2_ with a hexagonal structure, Z = 2.

From the available crystallographic data of Ti_4_S_2_C_2_ and Ti_2_S_2_ [9,10,11,18,28], the Ti_4_S_2_C_2_ and Ti_2_S_2_ crystallize in the hexagonal space group P63/MMC (space group No. 194) with two formula units (Z = 2) per cell, as shown in Figure 8. When one S atom in the Ti_2_S_2_ cell was replaced by a C atom, Ti_2_SC was formed and the structural properties of Ti_2_SC was calculated and predicted. In order to analyze the phase stability, TiC with NaCl structure (Z = 4, space group No. 192) [29] was also calculated.

Through the total energy, calculated as a function of volume, the ground state properties of these phases are analyzed. It is possible to obtain the equilibrium lattice constants of these phases from the Brich-Murnaghan equation of state [30,31], (Table 1). The calculation lattice constants match well with the experimental ones for Ti_2_S_2_ [28] and Ti_4_C_4_ [29]. The difference between the theoretical and experimental values for Ti_4_S_2_C_2_ is obvious. The main reason for this may be the effects of early measurement accuracy or compound purity. Unfortunately, we have no recent experimental data for Ti_4_S_2_C_2_. Based on the data of Ti_2_S_2_ and TiC (see Table 1), the calculation accuracy in this work is reliable. From Table 1, the c/a value of Ti_4_S_2_C_2_ can be seen to be greater than that of Ti_2_SC, so the structural stability of Ti_2_SC is higher than Ti_4_S_2_C_2_ in the view of the c/a.

The formation enthalpy (ΔH_f_) of Ti_x_S_y_C_z_ of the elements (hcp-Ti, rhombic sulfur and graphite) can be shown as:

(1) $$\Delta E_{f}=\frac{E\left({\mathrm{Ti}}_{x}S_{y}C_{z}\right)-\mathrm{xE}\left(\mathrm{Ti}\right)-\mathrm{yE}\left(S\right)-\mathrm{zE}\left(C\right)}{x+y+z}$$

The formation enthalpy equals the calculated formation energy (ΔE_f_), i.e., ∆H_f_ = ∆E_f_, when the zero-vibration contribution is ignored at T = 0 K and p = 0 Pa [32,33]. The formation energy can represent the thermo-stability of Ti_x_S_y_C_z_ (Table 1). The formation enthalpy of TiC in this work is close to the results of others [34,35,36]. The phases with negative formation energies are thermodynamically stable. The ΔE_f_ of Ti_2_S_2_ is −3.81 eV/f.u. (formula unit) and that of Ti_4_C_4_ is −1.88 eV/f.u. The ΔE_f_ of Ti_4_S_2_C_2_ (−2.12 eV/f.u.) is higher than [TiS] + [TiC] (−5.69 eV/f.u.), which shows that the Ti_4_S_2_C_2_ (Z = 2) is less stable than ([TiS] + [TiC]). The ΔE_f_ of Ti_2_SC (−6.70 eV/f.u.) is more negative than [TiS] + [TiC] (−5.69 eV/f.u.), which indicates that the Ti_2_SC is more stable than ([TiS] + [TiC]). That is to say, Ti_2_SC does not spontaneously decompose into TiS and TiC; however, Ti_4_S_2_C_2_ (Z = 2) can spontaneously change into the two phases.

### 3.5. Electronic Structure of Phases

**Figure 9:**
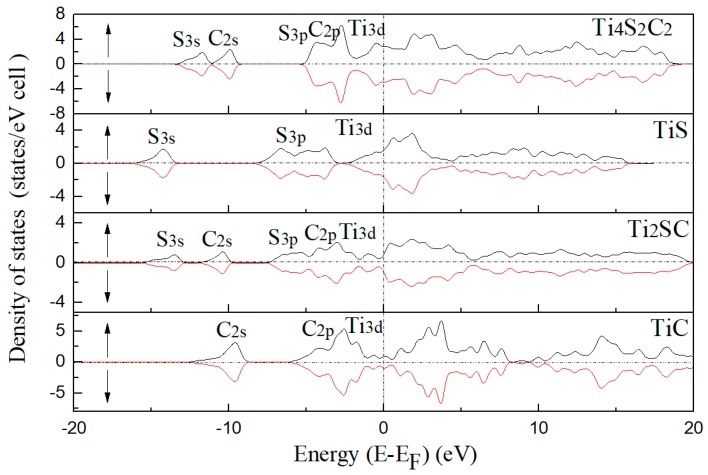
The total density of the states Ti_4_S_2_C_2_, Ti_2_S_2_, Ti_2_SC and Ti_4_C_4._

The electronic structures will be compared with each other. Figure 9 describes the spin-projected DOSs (density of states) for Ti_4_C_4_ (Z = 4), Ti_4_S_2_C_2_ (Z = 2), Ti_2_SC and Ti_2_S_2_ (Z = 2). There are no energy gaps near the Fermi level. This indicates that these compounds are with the metallic nature. From the lowest valence band to the upper valence band, there are 3.75, 4.65, 1.98 and 2.37 eV energy gaps for Ti_4_S_2_C_2_ (Z = 2), Ti_2_S_2_ (Z = 2), Ti_2_SC and Ti_4_C_4_ (Z = 4), respectively. It can be concluded that the chemical bonds of these compounds take on ionicity and the ionicity of Ti–S is stronger than Ti–C. For Ti_4_S_2_C_2_ (Z = 2), the sulfur 3s band mainly lies between −13.6 and −11 eV, the carbon 2s band mostly ranges from −11 to −9.4 eV, and the sulfur 3p band and carbon 2p band mostly range from −5.3 to the Fermi level. A similar DOS of Ti_4_S_2_C_2_ (Z = 2) was given by Ramalingam et al. [37]. For Ti_2_S_2_ (Z = 2), the sulfur 3s band mainly lies between −16.2 and −13.2 eV, and the sulfur 3p band mostly ranges from −8.5 to the Fermi level. For Ti_2_SC, the sulfur 3s band mainly lies between −15.7 and −12.6 eV, the carbon 2s band mostly ranges from −12 to −9.5 eV, and the sulfur 3p band and carbon 2p band mostly range from −7.5 eV to the Fermi level. For Ti_4_C_4_ (Z = 2), the carbon 2s band mainly lies between −12.7 and −8.5 eV, and the carbon 2p band mostly ranges from −6.1 eV to the Fermi level. The DOS of TiC in this work is consistent with that in the literature [34]. The 2s and 3p energy bands of sulfur obviously shift to a high-energy zone for Ti_4_S_2_C_2_ (Z = 2), with a shift that is slight in Ti_2_SC. For these compounds, there is obvious hybridization between S/C p electron bands and Ti 3d electron bands, which shows the covalent interaction between C/S and Ti. The chemical bonds of these compounds take on ionicity and the ionicity of Ti–S is stronger than Ti–C.

**Figure 10:**
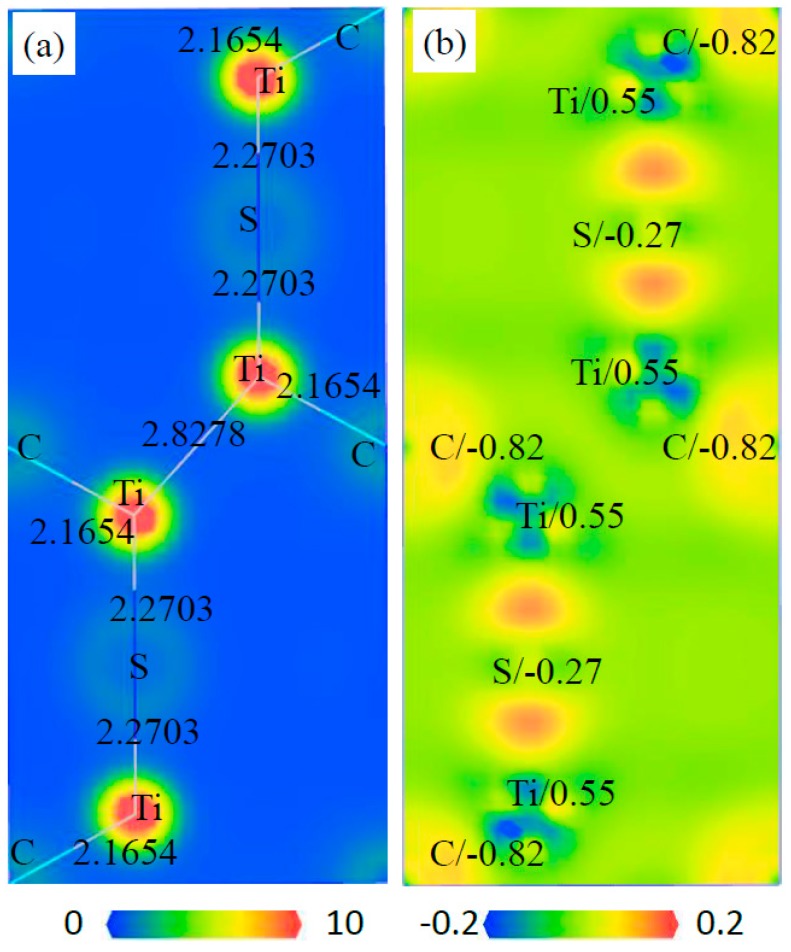
Electron density maps of the (110) plane in Ti_4_C_2_S_2_, (**a**) total electron density map from 0 to 10 (e/Å^3^) and the bond length (Å), (**b**) electron density difference map (e/Å^3^) and the atomic populations (e).

**Figure 11:**
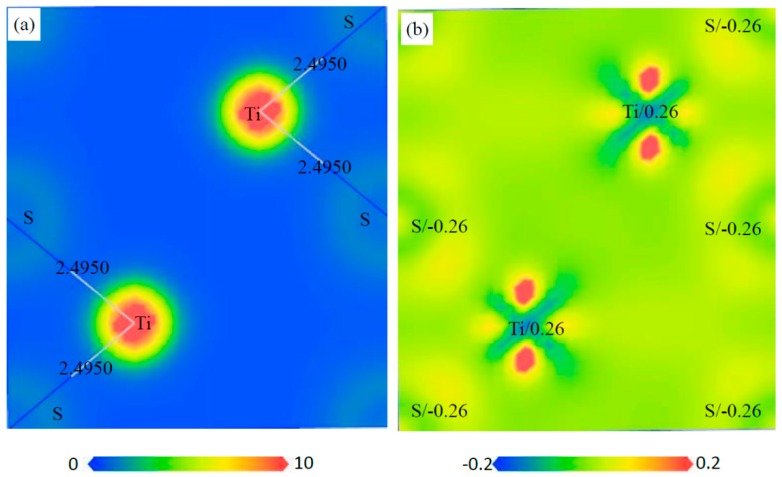
Electron density maps of the (110) plane in Ti_2_S_2_, (**a**) total electron density map from 0 to 10 (e/Å^3^) and the bond length (Å), (**b**) electron density difference map (e/Å^3^) and the atomic populations (e).

**Figure 12:**
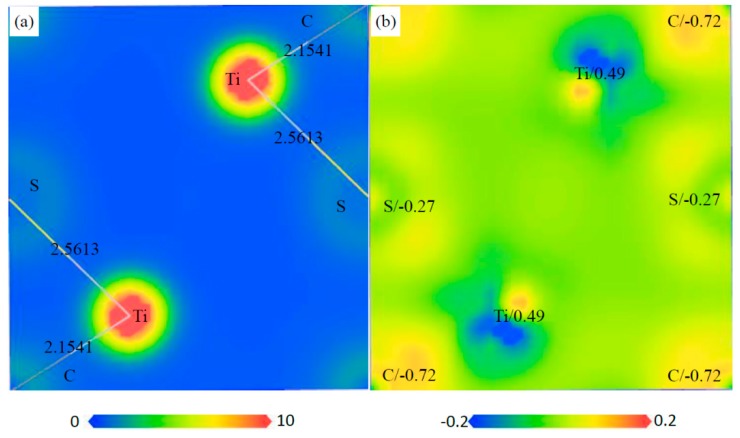
Electron density maps of the (1 1 0) plane in Ti_2_SC, (**a**) total electron density map from 0 to 10 (e/Å^3^) and the bond length (Å), (**b**) electron density difference map (e/Å^3^) and the atomic populations (e).

**Figure 13:**
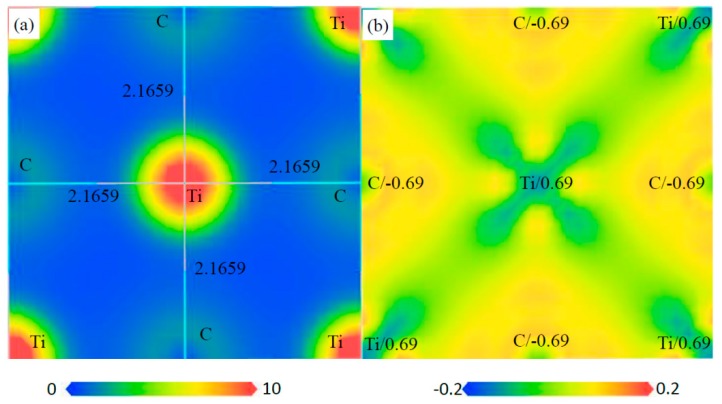
Electron density maps of the (0 0 1) plane in Ti_4_C_4_, (**a**) total electron density map from 0 to 10 (e/Å^3^) and the bond length (Å), (**b**) electron density difference map (e/Å^3^) and the atomic populations (e).

The charge distribution and transfer of atoms within a compound can be characterized by the electron density distributions and Mulliken population analysis. The electron density distribution maps are plotted in two ways: the total electron density and electron density difference maps, as shown in Figure 10, Figure 11, Figure 12 and Figure 13. These total electron density distributions are similar to each other, with the densities around the Ti and S/C sites being almost spherical and the core regions of Ti having the largest density. 

The length of Ti–C is 2.1654 Å and that of Ti–S is 2.2703 Å in Ti_4_C_2_S_2_. The length of Ti–C is 2.1659 Å in Ti_4_C_4_ and that of Ti–S is 2.4950 Å in Ti_2_S_2_. The length of Ti–C is 2.1541 Å and that of Ti–S is 2.5613 Å in Ti_2_CS. It can be seen that the Ti–C bond in Ti_2_CS is a little shorter than that in Ti_4_C_4_, and the Ti–S bond is a little longer than that in Ti_2_S_2_. However, the Ti–C bond in Ti_4_C_2_S_2_ is close to that in Ti_4_C_4_, and the Ti–S bond is shorter than that in Ti_2_S_2_ by about 10%. This indicates that Ti–S bonds are compressed in Ti_4_S_2_C_2_ cells, which may lead to poor structural stability for Ti_4_S_2_C_2_.

The electron density difference can be determined from the equation: $\Delta \rho =\left\{\rho_{\mathrm{crystal}}-\sum \rho_{\mathrm{at}}\right\}$ [38], where $\rho_{\mathrm{crystal}}$ and $\rho_{\mathrm{at}}$ are the electron densities of these crystals (i.e., Ti_4_S_2_C_2_, Ti_2_S_2_, Ti_2_SC and Ti_4_C_4_) and the corresponding free atoms, respectively. The transfer of electrons in space can be from reflected from the electron density difference maps. The electrons of Ti atoms decrease and those of S/C atoms increase in these phases, so an exchange of electrons takes place between Ti and S/C atoms. The charge of each atom can be calculated from the Mulliken method, and the calculated charges of Ti and S/C atoms are different in the various phases. The calculated charges of Ti, S and C are 0.55, −0.27 and −0.82 in Ti_4_S_2_C_2_, respectively. The calculated charges of Ti and S are 0.26 and −0.26 in Ti_2_S_2_, respectively. The calculated charges of Ti, S and C are 0.49, −0.27 and −0.72 in Ti_2_SC, respectively. The calculated charges of Ti and C are 0.69 and −0.69 in Ti_4_C_4_, respectively. This further illustrated the electron transfer from Ti to S/C atoms in these phases.

### 3.6. Elastic Properties of Phases

The strain energies of a crystal are positive, which implies that the crystal has mechanical stability. The elastic constants C_ij_ depending on the crystal structure can describe the mechanical stability of the crystal. The elastic constants were calculated using the stress-strain method after obtaining equilibrium geometry [38,39]. The elastic constants of Ti_4_S_2_C_2_, Ti_2_S_2_, Ti_2_SC and Ti_4_C_4_ are shown in Table 2.

The calculated values of Ti_4_C_4_ are similar to the results of the literatures [34,35,40]. For the hexagonal crystal structures (Ti_4_S_2_C_2_, Ti_2_S_2_ and Ti_2_SC), there are five different symmetry elements (C_11_, C_12_, C_13_, C_33_, C_44,_). The mechanical stability criteria for a hexagonal crystal are [38,39]:

C_11_ > 0, C_11_ − C_12_ > 0, C_44_ > 0, (C_11_ + C_12_)C_33_ − 2C_13_^2^ > 0 (2)

Cubic crystal structures (Ti_4_C_4_) have three independent symmetry elements (C_11_, C_12_, and C_44_). The stability criteria for a cubic crystal are [41]:

C_11_ > 0, C_11_ − C_12_ > 0, C_44_ > 0, C_11_ + 2C_12_ > 0 (3)

From Table 2, Ti_2_S_2_, and Ti_4_C_4_ are mechanically stable, because their elastic constants satisfy the stability criteria. However, the elastic constants of Ti_4_S_2_C_2_ do not satisfy the stability criteria. The mechanical stability of Ti_2_SC is higher than that of Ti_4_S_2_C_2_ form the calculation results, which is in agreement with the analysis of lattice parameters, as the c/a value of Ti_4_S_2_C_2_ (4.05) is bigger than that of Ti_2_SC (1.85). 

If it cannot obtain single crystal samples, measuring the individual elastic constants C_ij_ is impossible. The polycrystalline elastic moduli can be directly calculated by these elastic constants using the Voigt-Reuss-Hill (VRH) method. Instead, the isotropic bulk modulus B and shear modulus G are determined [42]. By using the equation (4) [43,44], the universal anisotropy index (A_U_) was calculated.

(4) $$A_{U}=5\frac{G_{V}}{G_{R}}+\frac{B_{V}}{B_{R}}-6$$

The isotropic Young’s modulus E (Gpa), shear modulus G, bulk modulus B, Poisson’s ratio ν, universal anisotropy index (A_U_), and Debye temperature (θ_D_ in K) are shown in Table 3. The calculated mechanical properties of Ti_4_C_4_, such as B, G and θ_D_ etc., agree with literatures [34,35,36].

The Zener anisotropy ratio, A_U_, indicates the degree of elastic anisotropy, and it takes the value of zero for a completely isotropic material. The anisotropic degree of materials increases with an increasing A_U_ value. From Table 3, the A_U_ value of Ti_4_C_4_ (Z = 4) is very low (0.01), close to zero, which indicates that the mechanical properties of this phase are approximatively isotropic. The A_U_ of Ti_2_SC is bigger than Ti_2_S_2_ and Ti_4_C_4_, so the anisotropic degree of Ti_2_SC is higher than that of the other two phases.

The ratio of B/G can estimate the ductility of a crystal, with increasing ductility corresponding to an increasing B/G ratio. The critical value of B/G is 1.75, which is separating ductile and brittle behavior of a material [41,45]. The ratios (B/G) of Ti_2_S_2_, Ti_2_SC and Ti_4_C_4_ decrease with an increasing C. The B/G ratios of Ti_4_C_4_ (Z = 4) and Ti_2_SC are lower than the critical value (1.75), which indicates that they are hard brittle phases. The B/G ratios of Ti_2_S_2_ (Z = 2) is higher than the critical value 1.75, which indicates that Ti_2_S_2_ (Z = 2) is a ductile phase. These hard brittle phases are difficult to undergo plastic deformation and are usually broken, which is in agreement with the microstructural observation of Y phases.

## 4. Conclusions

Y phases in AISI 321 stainless steel are like curved strips or bones, which mostly show clustered distribution and are approximately arranged in parallel. The width of Y phases mainly ranges from 0.5 to 2 μm; however, the length is from a few microns to tens of microns. The EDS analysis of the Y phase shows that the composition of Y phase is close to Ti_2_SC. After homogenizing annealing (1290 °C for 24 h), there is no significant change in the distribution and metallographic characteristics of the Y phase, which indicates that Y phases exhibit high thermal stability. Y phases are broken and fragmented during the forging process, especially in the case of longer ones. The degree of fragmentation increases with an increase in the forging deformation for Y phases and the sizes of Y phases decrease with increasing the deformation. The number of smaller Y phases increase, as the number of the bigger ones decrease. With the increase in deformation, the values of length and width are close to each other. From first calculations, the Ti_2_SC does not spontaneously change into TiS and TiC; however, Ti_4_S_2_C_2_ (Z = 2) can spontaneously change into the two phases. The Ti–S bonds are compressed in Ti_4_S_2_C_2_ cell, which may lead to poor structural stability for Ti_4_S_2_C_2_. The chemical bonds of these compounds take on ionicity and the ionicity of Ti–S is stronger than Ti–C. In these compounds, there is obvious hybridization between S/C p electron bands and Ti 3d electron bands, which shows covalent interactions between C/S and Ti. There is an exchange of electrons that takes place between Ti and S/C atoms. Evidently, the mechanical stability of Ti_4_S_2_C_2_ is poor, while Ti_2_SC has high structural stability. The ratios (B/G) of Ti_2_S_2_, Ti_2_SC and Ti_4_C_4_ decrease with increasing C. Ti_2_S_2_ (Z = 2) is a ductile phase, while Ti_2_SC is a hard brittle phase. The hard brittle phase Ti_2_SC does not easily undergo plastic deformation and is usually broken.

## Figures and Tables

**Table 1:** Calculated lattice constants, c/a, volume, formation enthalpy for unit cell ∆E_f_-cell (eV/cell), formation enthalpy for formula unit (f.u.) ∆E_f_-f.u. (eV/f.u.) and formation enthalpy ∆E_f_ (eV/atom) of the phases.

Phase	a (Å)	b (Å)	c (Å)	c/a	V_cell_ (Å^3^)	∆E_f_-cell	∆E_f_-f.u.	∆E_f_
Ti_4_S_2_C_2_(Z = 2)	3.2802	3.2802	13.2810	4.05	123.752	−8.46	−2.12	−1.06
Ti_2_S_2_(Z = 2)	3.3342	3.3342	6.3494	1.90	61.13	−7.61	−3.81	−1.90
Ti_2_SC	3.1702	3.1702	5.8550	1.85	50.96	−6.70	−6.70	−1.46
Ti_4_C_4_(Z = 4)	4.3317	4.3317	4.3317	-	81.28	−7.54	−1.88	−0.94

**Table 2:** Elastic constants of C_ij_ (GPa) of phases.

Phase	C_11_	C_12_	C_13_	C_33_	C_44_
Ti_4_S_2_C_2_ (Z = 2)	154.4	16.4	71.0	419.1	−23.2
Ti_2_S_2_ (Z = 2)	195.7	82.5	99.5	194.3	70.4
Ti_2_SC	328.3	79.6	82.4	348.2	86.1
Ti_4_C_4_(Z = 4)	516.6	115.1	-	-	182.8

**Table 3:** Calculated Young’s modulus E (GPa), bulk modulus B (GPa), shear modulus G (GPa), Poisson’s ratio (ν), universal anisotropy index (A_U_), and Debye temperature (θ_D_ in K) of the phases.

Phase	E	B	G	ν	A_U_	θ_D_
Ti_4_S_2_C_2_ (Z = 2)	−111.4	100.4	−33.1	-	-	-
Ti_2_S_2_ (Z = 2)	153.2	127.5	58.9	0.300	0.146	492
Ti_2_SC	265.5	165.9	107.6	0.233	0.224	684
Ti_4_C_4_ (Z = 4)	454.9	248.9	189.8	0.196	0.011	944
